# Effects of Comedication and Genetic Factors on the Population Pharmacokinetics of Lamotrigine: A Prospective Analysis in Chinese Patients With Epilepsy

**DOI:** 10.3389/fphar.2019.00832

**Published:** 2019-07-25

**Authors:** Zhan-zhang Wang, Yue-feng Zhang, Wen-can Huang, Xi-pei Wang, Xiao-jiao Ni, Hao-yang Lu, Jin-qing Hu, Shu-hua Deng, Xiu-qing Zhu, Huan-shan Xie, Hong-zhen Chen, Ming Zhang, Chang Qiu, Yu-guan Wen, De-wei Shang

**Affiliations:** ^1^Department of Pharmacy, The Affiliated Brain Hospital of Guangzhou Medical University (Guangzhou Huiai Hospital), Guangzhou, China; ^2^Guangdong Engineering Technology Research Center for Translational Medicine of Mental Disorders, Guangzhou, China; ^3^Department of Neurology, The Affiliated Brain Hospital of Guangzhou Medical University (Guangzhou Huiai Hospital), Guangzhou, China; ^4^Department of Pharmacy, Guangzhou Bureau of Civil Affairs Psychiatric Hospital, Guangzhou, China; ^5^Medical Research Center, Guangdong Province People’s Hospital, Guangdong Academy of Medical Sciences, Cardiovascular Institute, Guangzhou, China

**Keywords:** lamotrigine, Chinese, population pharmacokinetics, NONMEM, epilepsy

## Abstract

Lamotrigine (LTG) is a second-generation anti-epileptic drug widely used for focal and generalized seizures in adults and children, and as a first-line medication in pregnant women and women of childbearing age. However, LTG pharmacokinetics shows high inter-individual variability, thus potentially leading to therapeutic failure or side effects in patients. This prospective study aimed to establish a population pharmacokinetics model for LTG in Chinese patients with epilepsy and to investigate the effects of genetic variants in uridine diphosphate glucuronosyltransferase (UGT) 1A4, UGT2B7, MDR1, ABCG2, ABCC2, and SLC22A1, as well as non-genetic factors, on LTG pharmacokinetics. The study population consisted of 89 patients with epilepsy, with 419 concentrations of LTG. A nonlinear mixed effects model was implemented in NONMEM software. A one-compartment model with first-order input and first-order elimination was found to adequately characterize LTG concentration. The population estimates of the apparent volume of distribution (V/F) and apparent clearance (CL/F) were 12.7 L and 1.12 L/h, respectively. The use of valproic acid decreased CL/F by 38.5%, whereas the co-administration of rifampicin caused an increase in CL/F of 64.7%. The CL/F decreased by 52.5% in *SLC22A1*-1222AA carriers. Patients with the *ABCG2*-34AA genotype had a 42.0% decrease in V/F, whereas patients with the *MDR1*-2677TT and C3435TT genotypes had a 136% increase in V/F. No obvious genetic effect of UGT enzymes was found relative to the concentrations of LTG in Chinese patients. Recommended dose regimens for patients with different gene polymorphisms and comedications were estimated on the basis of Monte Carlo simulations and the established model. These findings should be valuable for developing individualized dosage regimens in adult and adolescent Chinese patients 13–65 years of age.

## Introduction

Lamotrigine (LTG), a second-generation anti-epileptic drug belonging to the phenyltriazine family, has been widely used for treating focal and generalized seizures and bipolar disorder type I in adults and children, either as a monotherapy (>12 years) or in combination with other anti-epileptic drugs (AEDs) (>2 years) (He et al., 2012). However, high inter-individual variability (IIV) of LTG pharmacokinetics has been observed in patients, thus potentially leading to therapeutic failure at supertherapeutic drug concentrations, or to decreased tolerability or adverse effects at supratherapeutic drug concentrations. In the latest Arbeitsgemeinschaft für Neuropsychopharmakologie und Pharmakopsychiatrie guideline (AGNP), therapeutic drug monitoring is recommended for LTG in cases involving dose titration or special indications (Hiemke et al., 2018).

LTG exhibits first-order linear pharmacokinetics with rapid and complete absorption when administered orally and is bio-transformed mainly through uridine diphosphate glucuronosyltransferase (UGT) (Chan et al., 2001), whose metabolism is significantly influenced by patient comedications. The co-administration of enzyme inhibitors [such as valproic acid (VPA) (Rivas et al., 2008; Brzakovic et al., 2014; Zhang et al., 2017; van Dijkman et al., 2018)] prolongs the LTG half-life by two-fold (van Dijkman et al., 2018), whereas the half-life is substantially shortened by coadministration of enzyme inducers [such as phenytoin (PHT) (Rivas et al., 2008; Brzakovic et al., 2014; Milosheska et al., 2016; Zhang et al., 2017; van Dijkman et al., 2018)].

Reports have shown that genetic polymorphisms of UGT significantly influence LTG exposure in patients. The overexpression of ATP-binding cassette sub-family B member 1 (ABCB1; MDR1; P-glycoprotein 170) and breast cancer resistance protein (BCRP; ABCG2), major human efflux transporters for LTG at the blood–brain barrier, have been shown to be important mechanisms of pharmacoresistance in patients with epilepsy (Romermann et al., 2015). Efforts have been made to investigate the effects of MDR1 and ABCG2 transporters’ polymorphism on LTG pharmacokinetics in humans (Lovric et al., 2012; Zhou et al., 2015; Shen et al., 2016; Domjanovic et al., 2018). *MDR1* rs1128503 and rs1045642 and *ABCG2* rs2231142, rs3114020, and rs2231137 have been found to be associated with the disposition of LGT (Lovric et al., 2012; Zhou et al., 2015; Shen et al., 2016). However, some of these findings are conflicting and require further investigation (Shen et al., 2016; Domjanovic et al., 2018). The organic cation transporter 1 (OCT1, encoded by the *SLC22A1* gene), expressed primarily in epithelial cells, also suggests a major role of the membrane transporter in the pharmacokinetics of many organic cationic compounds (Arimany-Nardi et al., 2015). Recently, OCT1 has been implicated in the transport of LTG both into hepatocytes and at the blood–brain barrier (Dickens et al., 2012). The genetic effects of *OCT1* polymorphisms on responses and adverse events have been found to be ambiguous and to potentially be population-specific (Mato et al., 2018). Among the known polymorphisms of the *SLC22A1* gene, *SLC22A1* rs628031 is among the most studied. In the one study on Chinese patients with epilepsy, a statistically significant association between *SLC22A1* rs628031 polymorphism and normalized LTG concentration has been observed (Shen et al., 2016). However, for other drugs transported by OCT1, there are contrasting reports regarding the roles of rs628031 polymorphisms in the treatment response or drug-related adverse effects (Tarasova et al., 2012; Koren-Michowitz et al., 2014; Cargnin et al., 2018). The studied single nucleotide polymorphisms (SNPs) for *SLC22A1* are also limited in Chinese people, and more research is needed to obtain a solid basis to design personalized therapeutic approaches.

Population pharmacokinetic analysis has previously been applied to drugs with high IIV (Sheng et al., 2016). The influence of comedication with enzyme modifiers on LTG has been quantified through a population approach in several studies in adults and in children (Chan et al., 2001; Siddiqui et al., 2003; Liu et al., 2015; Milosheska et al., 2016). However, population pharmacokinetic studies including all genetic polymorphisms of transporters and enzymes and patient characteristics are limited. One study in Caucasian adult patients has investigated the effects of genetic polymorphisms of *SLC22A1*, *UGT2B7*, *UGT1A4*, and *ABCB1* on LTG concentration and has included *UGT2B7* polymorphisms, coadministration of enzyme inducers, smoking status, and body weight in the final model (Milosheska et al., 2016). Another study in Thai people has investigated the effects of genetic variants of *UGT1A4* and *UGT2B7*, as well as non-genetic factors, on LTG pharmacokinetics and has found that enzyme inducers, VPA, and the *UGT2B7*-161 C > T SNP significantly influence LTG clearance (Singkham et al., 2013). Although two population pharmacokinetic (PPK) LTG studies have been conducted to identify non-genetic factors in Chinese children (Liu et al., 2015; Zhang et al., 2017), the identification and quantification of genetic polymorphisms in Chinese children and adults, especially with complicated comedication with enzyme modifiers in real-world therapy, are still lacking.

The aim of this clinical study was to characterize the PPK in child and adult Chinese patients with epilepsy, to confirm known enzyme modifiers [e.g., VPA and rifampicin (RFP)], and to identify more potential genetic predictors of LTG PPK. The results presented here may guide dosing for individual patients.

## Subjects and Methods

### Patients and Study Design

This prospective study was conducted in out-patients with epilepsy who received LTG titration treatment during ambulatory visits between July 2014 and January 2017 at Guangzhou Huiai Hospital, China. All enrolled patients were previously informed about the purpose of the study and provided the written informed consent. The study was approved by the Medical Ethics Committee of Guangzhou Huiai Hospital and was carried out according to the Declaration of Helsinki.

Patients taking LTG orally either as a monotherapy or as combination therapy were enrolled. Demographic data (age, weight, height, and sex), smoking status, adverse event data, duration of current therapy, concomitant drug therapy (such as RFP and VPA), comorbidities, and LTG dosing regimens were determined throughout the study. In patients < 18 years of age, the dose was based on body weight. Comedication status was recorded when the LTG concentration was determined during the study. Biochemical parameters, including serum creatinine, aspartate aminotransferase, and alanine transaminase, were also obtained from the patient charts. Patients were asked to record the time of the last dose before the ambulatory visit.

One blood sample per patient was collected with EDTA-Na as an anticoagulant at the beginning of the study. The blood cells were collected and stored at −70°C for genotyping analysis. Procoagulant blood samples for serum concentration measurements were drawn sparsely at steady state (after at least 7 days on the same dose), mainly at 0.5 and 1 h prior to the next dosing. The procoagulant blood samples were centrifuged at 3,000 *g* for 10°min. Serum for LTG measurement was collected and stored at −70°C until drug analysis.

### Bioanalysis

The serum concentrations of LTG were determined through a validated high-performance liquid chromatography-electrospray ionization tandem mass spectrometry (HPLC-MS/MS) method (Serralheiro et al., 2013) performed on an Agilent 1200 liquid chromatography model integrated system connected to an Agilent 6410 Mass Spectrometer (Agilent Technologies Inc., Santa Clara, CA, USA). An isotope of LTG (LTG-^13^C_3_, purity 99%, TRC Ltd., Toronto, Canada) was used as the internal standard. Protein precipitation with 500 μl methanol was used to extract LTG from 100 μl serum. The extracted samples were separated on an Agilent Eclipse plus C_18_ (4.6 × 100 mm, 3.5 μm) with the mobile phase consisting of methanol:5 mM ammonium formate solution (85:15, *V:V*). The calibration curves were linear over the range of 0.20–25.00 µg × ml^–1^, and the lower limit of quantification was validated at 0.20 µg × ml^–1^. Recovery was examined at three different concentrations and was found to be above 98.4%. The intra- and inter-day precision, as reﬂected by the coefficient of variation, was below 2.31%, whereas the accuracy was within 95.9–111.8% of the nominal values.

### Genotyping Analysis

The genotypes of all patients were determined. Genomic DNA was extracted from peripheral blood leukocytes through the phenol-chloroform method. The genotyping of *UGT1A4* 70C > A (rs6755571) and 142T > G (rs2011425), *ABCB1* C1236T (rs1128503), G2677T (rs2032582), and C3435T (rs1045642) was performed through PCR-sequencing. The genotyping of *UGT2B7* 256-430C > T (rs4356975), *UGT2B7* 161C > T (rs7668258), *UGT2B7* 372A > G (rs7662029), *UGT2B7* 900G > A (rs7438135), *ABCG2* 34G > A (rs2231137), *ABCG2* 421C > A (rs2231142), *ABCC2* 1249G > A (rs2273697), *SLC22A1* 1222G > A (rs628031), and *SLC22A1* 1022C > T (rs2282143) was performed through the SNaPshot mini-sequencing method (Vivanti et al., 2019). All genotype determinations were performed in BGI-Shenzhen (Shenzhen, China). The genotype distributions were evaluated for Hardy–Weinberg equilibrium with a χ^2^ test with Yates’ correction applied. The threshold for statistical significance was set at *P* < 0.05. Statistical Package for the Social Sciences (SPSS, Chicago, IL, USA) ver.13.0 was used for analyses.

### Population Pharmacokinetic Modeling

The plasma concentrations from both sparse and rich datasets were analyzed through nonlinear mixed-effects modeling with NONMEM, version 7, level 3.0 (Icon Development Solutions, Ellicott City, MD, USA).

One-compartment model with first-order absorption and elimination was evaluated to fit the concentration *versus* time data. The absorption rate constant (K_a_) was fixed to the previously reported value of 1.97 h^−1^ because of the lack of serum samples around the absorption phase (Milosheska et al., 2016). The first-order conditional estimation method with interaction was used to obtain PPK parameters (θ) and their variability (η). The IIV was modeled with an exponential error model assuming a normal distribution with a mean of zero and variance of ω_p_^2^. Additive, proportional and combined additive/proportional error models were evaluated separately to account for the residual variability (ε) between the observed and predicted concentrations (variability not attributed to differences in dose regimen).

The covariates of age, sex, body weight, renal function, smoking, combination therapy, and genotypes of *UGT1A4*, *UGT2B7*, *MDR1*, *ABCG2*, *ABCC2*, and *SLC22A1* were analyzed in the model through a stepwise approach.

Discrete covariates (such as sex and comedications) were investigated with a fractional model (*P* *_ij =_* *P* *_tv_*_,_ *_j_*• (1 + θ*_j_* • *COV* *_dis_*)) in which discrete covariates such as sex or comedications (e.g., VPA or RFP) were incorporated by using dummy variables (*COV* *_dis_* = 0 for female, *COV* *_dis_* = 1 for male; *COV* *_dis_* = 1 if the patient was receiving certain concomitant drugs at the corresponding LTG sampling time point; and *COV* *_dis_* = 0 if the patient was not). Continuous covariates (such as weight and age) were introduced into the model in a linear manner as *P* *_ij =_* *P* *_tv_*_,_ *_j_* • (1 + θ*_j_* • (*COV* – *COV* *_ave_*)) • *e* ^η^ *^i^*, where *COV* is the continuous covariate, *COV* *_ave_* is the average value of the corresponding covariates, and θ*_j_* is a factor adjusting the *j*th PPK parameter. Genotypes were investigated through the use of dummy variables of different levels in a linear model, as *P* *_ij =_* *P* *_tv_*_,_ *_j_* • (1 + θ*_j_*), where each genotype (e.g., the wild-type homozygote, the heterozygote, and the mutant homozygote) could have a different θ*_j_* value; *P* *_tv_*_,_ *_j_* is the typical PPK value of the population without mutation, and θ*_j_* is a factor adjusting the *j*th PPK parameter.

The identification of significant covariates was performed through forward inclusion (for which an objective function value [ΔOFV] decrease in more than 3.84 is significant, *P* < 0.05, df = 1) and backward exclusion (for which a ΔOFV increase in more than 6.63 is significant, *P* < 0.01, *df* = 1). Goodness-of-fit plots, OFV values, accuracy and precision of parameter estimates, and random and residual variances for the parameters were also used to evaluate the significance of covariates and to validate the different models. The 95% confidence intervals of the parameters were evaluated through nonparametric bootstrapping without stratification (*n* = 1,000).

### Model Evaluation

The final model was evaluated with goodness-of-fit plots, the normalized prediction distribution error (NPDE), and external validation. The diagnostic plots were visualized with R (v. 2.12.2; Team R, 2010). One thousand simulations for NPDE were conducted with Perl-speaks-NONMEM (PsN) (v. 3.4.2). The NPDE plots were visualized with the R add-on packages Xpose4 (v. 4.3.2) and NPDE (version 2.0).

External validation was performed through retrospective therapeutic drug monitoring data from an additional 114 patients with 384 LTG serum concentrations. The validation data set did not overlap the initial dataset. Population predictions based on the final model by using the MAXEVAL = 0 option were compared against the observations. To assess the accuracy and precision of the predictions, we calculated and compared the mean prediction error and root-mean-squared error (Zhu et al., 2017).

### Simulations to Achieve Target Concentrations

By comparing the percentage improvements (Kagawa et al., 2014) and tolerability (Hirsch et al., 2004) in patients with different plasma LTG levels, we determined the reference ranges and laboratory alert level for LTG. In the consensus guidelines for TDM (Hiemke et al., 2018), the recommended therapeutic reference range for LTG as an anticonvulsant drug is 3–15 μg/ml, and the laboratory alert level is 20 μg/ml. In Chinese patients with epilepsy, the reference range has been suggested to be narrowed to 3–8 μg/ml (Liao et al., 2005). However, the reference ranges are given for the majority of epileptic patients, although some patients would inevitably maintain optimum therapeutic outcomes at concentration ranges below the lower limit or above the upper limit of the AGNP reference range.

Here, we assumed an optimal range of 3–5 μg/ml in some patients. We first simulated concentrations of 0, 0.5, and 1 h prior to the next dosing from the first dose to the steady state on the basis of a commonly used dose of 100 mg twice daily (b.i.d.) in the following six types of patients: those without comedications or gene mutations (ordinary patients), those co-medicated with VPA only, those co-medicated with RFP only, those carrying the *MDR1* 2677TT and 3435TT genotypes, those carrying the *ABCG2* 34AA genotype, or those carrying the *SLC22A1* 1222AA genotype. Then, several scenarios of different dose regimens for different types of patients were simulated to reach the target steady-state trough concentrations of 3–5 μg/ml, by using the final PPK model with typical parameters. The population predictions of LTG concentrations are shown for each simulation scenario.

## Results

### Patient Characteristics

A total of 419 LTG serum concentrations were collected from 89 subjects (42 males and 47 females) with a mean age of 28 years (range 4–63 years). A summary of the demographic and clinical characteristics of enrolled patients is shown in **Table 1**.

**Table 1:** Demographic characteristics of patients (range).

Patient characteristic	Value
Number of patients (*n*)	89
Gender M/F, *n* (%)	42 (47%)/47 (53%)
Age, year	28 (4–63)
Body weight, kg	59 (15–94)
Height, m	1.54 (11.5–18.3)
Body mass index, kg·m^-2^	25.1 (7.13–44.7)
Dose, mg	118 (6.25–300)
Duration of therapy, wk	42.8 (4–204)
No. of observations (obs.), *n* (%)	419
Lamotrigine alone	187 (44.6%)
Lamotrigine + VPA	157 (37.5%)
Lamotrigine + RFP	57 (13.6%)
Lamotrigine + both	19 (4.5%)

VPA, valproic acid; RFP, rifampicin. Data are presented as the mean with the range in parenthesis, or as the number of patients with the percentage in parenthesis.

### Frequencies of *UGTs*, *MDR1*, *ABBC2,* and *SLC22A1* Genotyping Variants

All DNA samples were able to be analyzed for all the polymorphisms present. There was no deviation from the expected proportions of genotypes in the population predicted according to Hardy–Weinberg equilibrium.

The distribution frequencies of the genotypes of the metabolic enzyme genes *UGT1A4* and *UGT2B7* in the groups of patients are listed in **Table 2**. The frequencies of the wild-type allele for *UGT1A4* (rs2011425) and *UGT2B7* (rs4356975, rs628031, rs28365063, and rs7438135) were higher than 48.3%, whereas the homozygous allele frequency was lower than 10.1%, in agreement with the frequencies previously reported in the Chinese population (Liu et al., 2015; Wang et al., 2015; Li et al., 2018). No patients with the *UGT1A4*-70C > A (rs6755571) mutation were found in the current study, which results similar to those in other studies on Asian [Japanese (Saeki et al., 2005) and Thai (Singkham et al., 2013)] populations.

The frequencies and distributions of the transporter genes *MDR1*, *ABCG2*, *ABCC2*, and *SLC22A1* are listed in **Table 3**. Regarding the *MDR1* gene, *MDR1*-C1236T showed a relatively high mutation rate of 33.7%, in agreement with a previous report (Ni et al., 2011). For the *MDR1*-G2677T polymorphism, 63 patients were heterozygous GG, GT, and TA carriers, and 12 patients (13.5%) were homozygous TT carriers (Ni et al., 2011). The mutation rates for *MDR1*-C3435T CC, CT, and TT were 47.2%, 38.2%, and 14.6%, respectively (Tan et al., 2005). Because the *MDR1*-G2677T and *MDR1*-C3435T mutations were dispersive and complex, we combined the two SNPs when building the covariate models to generate a combined covariate variable (*MDR1*-2677+C3435T). With respect to *ABCG2* 34G > A, carriers of the GG SNP accounted for 36.0% of all patients, and 13.5% of patients were homozygous for the mutation AA (Wan et al., 2015). The frequency of mutational homozygous AA variants for the *ABCC2* 421C > A polymorphism was relatively lower (5.6%), in agreement with findings from a previous study (Wan et al., 2015). AA homozygosity for *ABCC2*-1249AA was detected in 2.2% of patients, which was not seen in a previous study in the Chinese population (Zhang et al., 2008). Regarding the *SLC22A1* gene, AA homozygosity for *SLC22A1* 1222G > A was low but detectable in our current study (5.6%), similarl to findings in an Indian population study (Wan et al., 2015). Only two patients (2.2%) were determined to be AA carriers for the *SLC22A1*-1022G > A polymorphism in the current study, a number smaller than that among healthy subjects in Asian populations [10.77% in China, 9.68% in Malaysia, and 18.84% in India (Singh et al., 2012)].

**Table 2:** Allelic and genotype frequencies of the metabolism enzymes UGT1A4 and UGT2B7 in Chinese patients with epilepsy. (*n* = 89).

SNP	Genotype	Frequency (%)	Allele	Frequency (%)
*UGT1A4* 142T > G (rs2011425)	TT	55 (61.8)	T	139 (78.1)
	TG	29 (32.6)	G	39 (21.9)
	GG	5 (5.6)		
*UGT1A4* 70C > A (rs6755571)	CC	89 (100)	C	89 (100)
	CA	0 (0)	A	0 (0)
	AA	0 (0)		
*UGT2B7* 256-430C > T (rs4356975)	CC	68 (76.4)	C	148 (83.1)
	CT	12 (13.5)	T	30 (16.9)
	TT	9 (10.1)		
*UGT2B7* 161C > T (rs7668258)	CC	43 (48.3)	C	123 (69.1)
	CT	37 (41.6)	T	55 (30.9)
	TT	9 (10.1)		
*UGT2B7* 372A > G (rs7662029)	AA	60 (67.4)	A	146 (82.0)
	AG	26 (29.2)	G	32 (20.0)
	GG	3 (3.4)		
*UGT2B7* 900G > A (rs7438135)	AA	47 (52.8)	A	128 (71.9)
	AG	34 (38.2)	G	50 (28.1)
	GG	8 (9.0)		

**Table 3:** Allelic and genotype frequencies of the transporters MDR1, ABCG2, ABCC2, and SLC22A1 in Chinese patients with epilepsy. (*n* = 89).

Gene	Genotype	Frequency (%)	Allele	Frequency (%)
*MDR1*-C1236T (rs1128503)	CC	7 (7.9)	C	66 (37.1)
	CT	52 (58.4)	T	112 (62.9)
	TT	30 (33.7)		
*MDR1*-G2677T (rs2032582)	GG	22 (24.7)	G	89 (50.0)
	AT	8 (9.0)	A	24 (13.5)
	AG	12 (13.5)	T	65 (36.5)
	GT	33 (37.1)		
	AA	2 (2.2)		
	TT	12 (13.5)		
*MDR1*-C3435T (rs1045642)	CC	42 (47.2)	C	118 (66.3)
	CT	34 (38.2)	T	60 (33.7)
	TT	13 (14.6)		
*ABCG2* 34G > A (rs2231137)	GG	32 (36.0)	G	109 (61.2)
	AG	45 (50.6)	A	69 (38.8)
	AA	12 (13.5)		
*ABCG2* 421C > A (rs2231142)	CC	45 (50.6)	C	129 (72.5)
	CA	39 (43.8)	A	49 (27.5)
	AA	5 (5.6)		
*ABCC2*-G1249A (rs2273697)	GG	72 (80.9)	G	159 (89.3)
	GA	15 (16.9)	A	19 (10.7)
	AA	2 (2.2)		
*SLC22A1* 1222G > A (rs628031)	GG	41 (46.1)	G	125 (70.2)
	GA	43 (48.3)	A	53 (29.8)
	AA	5 (5.6)		
*SLC22A1* 1022C > T (rs2282143)	CC	72 (80.9)	C	159 (89.3)
	CT	15 (16.9)	T	19 (10.7)
	TT	2 (2.2)		

### Population Pharmacokinetic Modeling

**Figure 1:**
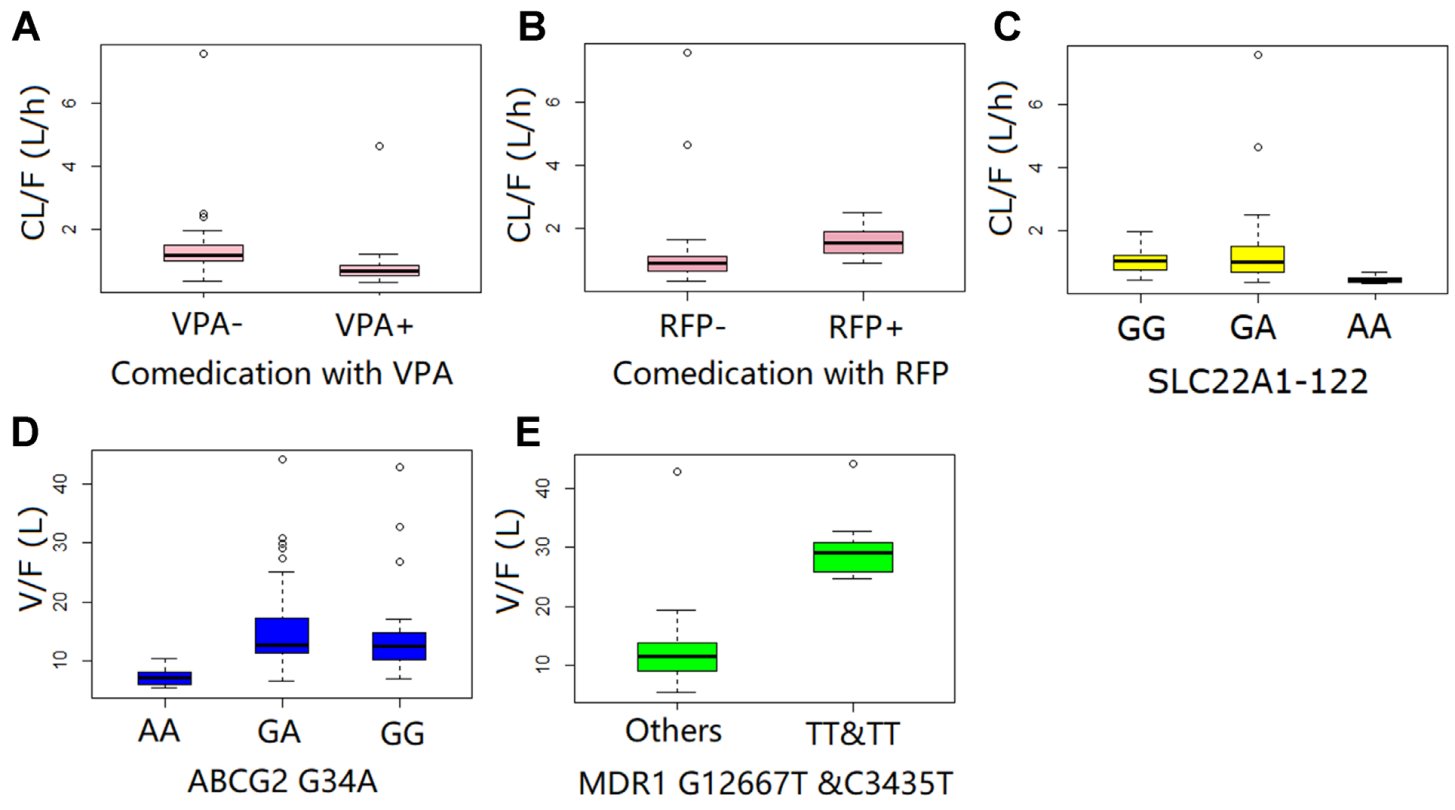
Diagnostic plots for the ﬁnal population pharmacokinetic model of lamotrigine, showing observed concentrations *vs*. population predicted **(A)** and individual predicted concentrations **(B)**. Conditional weighted residual error (CWRES) *vs*. population-predicted concentration of lamotrigine **(C)** and time after first dose **(D)**. Open circles represent individual data points. The solid line represents the unity line.

A one-compartment pharmacokinetic model with first-order absorption and proportional error adequately described the LTG PPK profiles. The parameter estimates of the primary and final PPK model are listed in **Table 4**. The first-order K_a_ for LTG was fixed at 1.97 h^−1^ according to a previously published pharmacokinetics study (Liu et al., 2015). A sensitivity analysis showed that when Ka was fixed to two-fold or half the current fixed value, the remaining parameter estimates were similar. Both IIV and error decreased in the final model. The decrease in the IIV on the basis of the apparent clearance (CL/F) and apparent volume of distribution (V/F) in the final model was 23% and 44%, respectively. Both polymorphisms of the transporters SNP *SLC22A1* 1222G > A and comedication with VPA and RFP were identified as covariates in the systemic CL/F of LTG. The population CL/F in patients without genetic mutations or comedications was 1.11 L/h. In *SLC22A1*-1222AA carriers, the CL/F was approximately 52.5% lower than that in non-carriers (*P* < 0.001). The co-administration of VPA caused a 38.5% decrease (*P* < 0.001) in CL/F, whereas the CL/F increased by 64.7% with the co-administration of RFP (*P* < 0.001). In contrast, the transporter polymorphisms *ABCG2*-34G > A, *MDR1*-C1236T, and *MDR1*-G2677T were identified as covariates in the V/F of LTG. The population V/F was 12.7 L in patients without the *ABCG2* 34G > A and *MDR1*-2677G-TT and C3435T-TT mutations. Patients with the *ABCG2*-34AA genotype showed a 42.0% decrease in V/F, whereas patients with *MDR1*-2677TT and C3435TT showed a 136% (2.36-fold) increase in V/F. Other covariates did not significantly influence the PPK parameters for LTG. The boxplots based on the various covariates are shown in **Figure 1**.

**Table 4:** Population pharmacokinetic parameter estimates of lamotrigine.

Parameters	Base model		Final model		
	Estimates	%CV	Estimates*	%CV	Bootstrap 95%CI
CL/F, L/h	0.959	11.4	1.12	14.6	0.95–1.52
V/F, L	13.6	23.8	12.7	28.4	9.57–20.38
K_a_, 1/h	1.97 FIX	–	1.97 FIX	–	–
θ_VPA on CL/F_	–	–	−0.386	19.1	−0.55–−0.25
θ_RFP on CL/F_	–	–	0.647	15.4	0.48–0.86
θ*_SLC22A1_*-_1222_ **_AA_**_ on CL/F_	–	–	−0.525	29.5	−0.79–−0.19
θ*_ABCG2_*_-34_ **_AA_**_ on V/F_	–	–	−0.420	35.5	−0.65−−0.14
θ*_MDR1_*_-2677_ **_TT_**_ + C3435_ **_TT_**_on V/F_	–	–	1.390	43.0	0.21–3.25
CL_INTER VAR_, %	22.6	73.5	15.2	113.2	2.47–47.89
V_INTER VAR_, %	29.1	115.8	20.1	172.6	1.00–98.95
Additive error, mg/L	1.20	41.0	0.797	31.0	0.28–1.35
Proportional error, %	21.8	22.4	0.167	24.4	10.24–20.77

CL/F, apparent system clearance; V/F, apparent distribution volume; K_a_, first-order absorption rate constant; θ, the factor of the covariate effect; INTER VAR, inter-individual variability; VPA, valproic acid; RFP, rifampicin; 95% confidence interval (95% CI) was calculated from the bootstrapping step (n = 1,000, success 929 times).

*The signs of covariate estimates represent trends of influence, that is, a plus sign refers to positive correlation and a minus sign refers to negative correlation.

The final model can be described by the following equations:

$$\text{CL}/\text{F}=1.12\times {\text{E}}_{\text{VPA}}\times {\text{E}}_{\text{RFP}}\times {\text{E}}_{\text{SLC}22\text{A}1-1222\text{AA}},$$

$$\text{V}/\text{F}=12.7\times {\text{E}}_{\text{ABCG}-34\text{AA}}\times E_{\text{MDR}1-2677\text{TT}+\text{C}3435\text{TT}},$$

where E_VPA_, E_RFP _, E_SLC22A1-1222_ **_AA_**, E_ABCG2-34_ **_AA_**, and E_MDR1-2677_ **_TT_**_ + C3435_ **_TT_** had values of 0.624, 1.647, 0.475, 0.680, and 2.390, respectively, for patients who were comedicated with VPA, comedicated with RFP, carriers of *SLC22A1*-1222AA, carriers of *ABCG2*-34AA, and carriers of *MDR1*-2677TT + C3435TT, respectively; otherwise, corresponding E value was assigned 1.

### Model Evaluation

**Figure 2:**
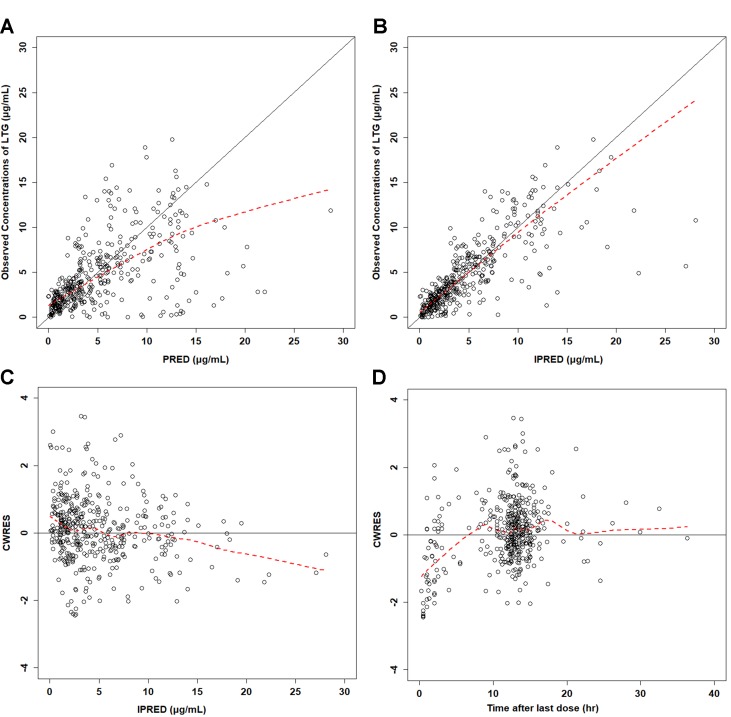
Boxplot of systematic clearance (CL/F) and distribution volume (V/F) of lamotrigine in different populations.

**Figure 3:**
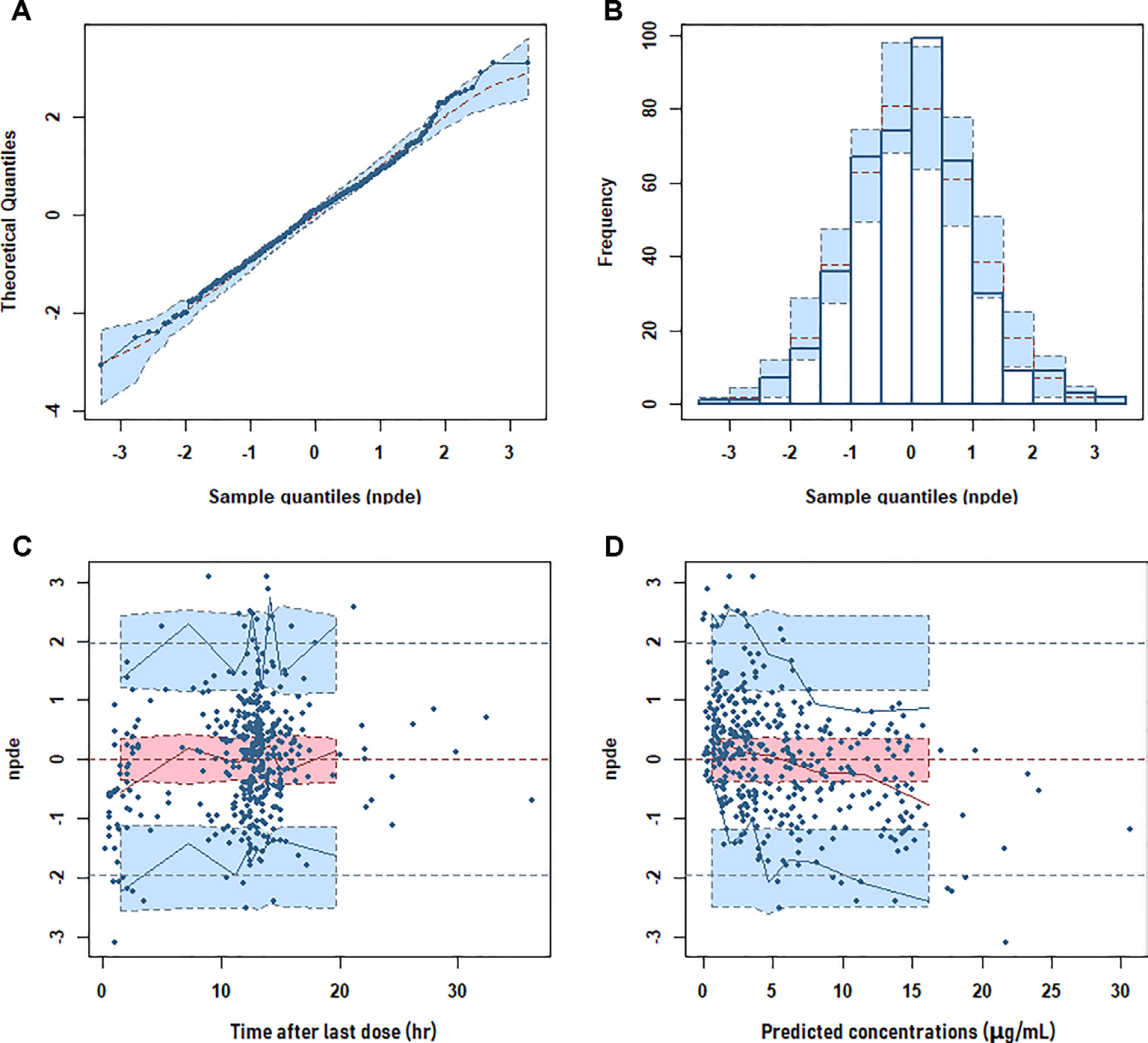
Normalized prediction distribution error (NPDE) metrics for the population pharmacokinetic model of lamotrigine. Normal quantile–quantile plot for NPDE **(A)**, distribution of NPDE **(B)**, and NPDE *vs*. time after first dose **(C)** or *vs*. predicted concentrations **(D)**.

The goodness of fit of the final population PK model is shown in **Figure 2**. The final PPK model was evaluated by using the NPDE method to validate the predictions of the model. Different dose regimens were used for each observation with 1,000 simulations (**Figure 3**). The quantile–quantile plot (**Figure 3A**) and the distribution histogram (**Figure 3B**) of NPDE had a mean of 0.0106 and a variance of 0.953, respectively (*p* = 0.504). There was no trend in NPDE *vs*. time (**Figure 3C**), but there was a slight trend in NPDE *vs*. predicted LTG concentration (**Figure 3D**) because of the sparse data in the high-concentration range. These results indicate that the population PK model of LTG is accurate and reliable.

In external validation, the bias (mean prediction error) was only 2.8 mg/L. The root-mean-squared error was 4.8 mg/L. The predicted concentrations were in good agreement with the observed concentrations without significant bias. The prediction errors were symmetrically distributed around zero and showed no significant patterns.

### Model Simulation

**Figure 4:**
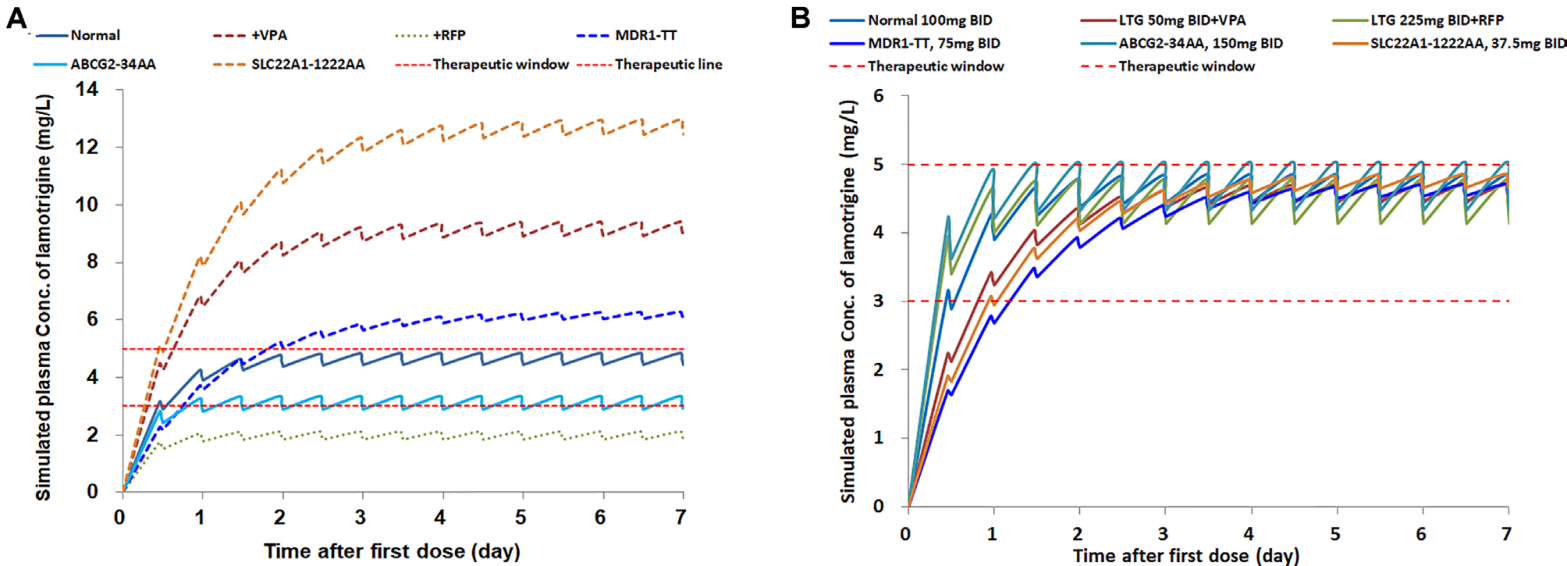
The simulations of lamotrigine pharmacokinetic profiles in patients, **(A)** with different concomitant drugs and genetic types, at the same dose of 100 mg b.i.d.; **(B)** with the same target concentration range, at personalized dose regimens.

According to the 2017 AGNP guidelines (Hiemke et al., 2018), the therapeutic reference range for LTG as an anticonvulsant drug is 3–15 μg/ml. The LTG exposure in the six types of patients is presented in **Figure 4A**, with the assumption that the optimum therapeutic range for certain individuals is 3–5 μg/ml, at a dose of 100 mg b.i.d. (normal dosage) (**Figure 4A**). Under the same dose regimen, only two types of patients were predicted to achieve the target steady-state concentration range (3–5 μg/ml): one with the *ABCG2*-34AA genotype and the other with no gene mutation or comedication with VPA or RFP, for which dosage adjustment might not be necessary. However, the comedication with RFP induced an obvious decrease in LTG exposure, which resulted in underexposure of LTG below the target level; comedication with VPA or the genotype *SLC22A1*-1222AA resulted in a large increase in LTG exposure up to ∼13 μg/ml, more than two-fold above the upper target level; *MDR1* TT carriers showed a slight increase in steady-state concentration. Because comedication and the *SLC22A1*-1222AA transporter mutation strongly influenced the exposure, dosage adjustments for these patients are suggested.

In the second scenario, for certain patients with an optimum therapeutic range of 3–5 μg/ml at a normal dosage of 100 mg b.i.d., the personalized dose regimens were applied on the basis of the covariates to achieve the target levels. The recommended dose range varied for different types of patients, from 37.5 mg to 225 mg b.i.d. The simulated PPK profiles of LTG indicated that the target steady-state concentration was achieved after continuous dosing for at least 3 days (**Figure 4B**). More rapid achievement of the target steady-state concentration was observed in the normal, ABCG2, and RFP groups within 2 days. Patients with the *SLC22A1*-1222AA genotype were given a dose (37.5 mg b.i.d). approximately one-third the normal dosage. Coadministration with RFP caused the dose to increase 2.25-fold over the normal dosage. The simulation results suggest that personalized therapy for LTG is necessary.

## Discussion

Several studies have considered the effects of age, weight, comedications (Chan et al., 2001; Rivas et al., 2008; Arif et al., 2011; Singkham et al., 2013; Brzakovic et al., 2014; Liu et al., 2015; Milosheska et al., 2016; Zhang et al., 2017; van Dijkman et al., 2018), UGT enzyme polymorphism (Singkham et al., 2013; Milosheska et al., 2016), and efflux transporters (Zhou et al., 2015) in the distribution and metabolism of LTG. However, most of these studies have not simultaneously included comedications and polymorphisms in enzymes and transporters. Our current study built a PPK model in Chinese patients and quantified the influences of genetic variants of metabolic enzymes and transporters, and non-genetic intrinsic and extrinsic factors on LTG pharmacokinetics. Polymorphisms of the transporter genes *SLC22A1*, *ABCG2*, and *MDR1* as well as comedications were identified as significant covariates for CL/F and V/F. In addition, the comedications administered in this prospective study were recorded and detailed as corresponding observations. Most of the patients included in the study were administered VPA or RFP for a short period of time, and the comedications changed during the entire therapy course, in line with actual clinical practice. This study thus generated a more systematic population division approach in modeling, making the estimation more accurate.

We attempted to include weight and age as covariates; however, neither showed important effects on the pharmacokinetics of LTG. van Dijkman et al. (2018) have detected sigmoidal changes in LTG clearance associated with age and have found that the clearance in children slowly decreases to adult values between the ages of 2 and 3 years. In addition, the estimated clearance in adults (0.0319 L/h/kg) is close to that in children and adolescents 2–18 years of age (0.0374 L/h/kg) (van Dijkman et al., 2018). Therefore, age was not likely to have influenced the pharmacokinetics in children (4–17 years) and adult patients (18–63 years) in our study. Moreover, the patient body weights were similar in adolescents and adults (**Supplementary Figure 1**), thus potentially partly explaining the nonsignificant effect of body weight observed herein. Given the small proportion of very young children included, our PPK model was suitable for patients 13–65 years of age.

Our results showed that despite the important roles of *UGT1A4* and *UGT2B7* polymorphisms in enzyme metabolic activity, the LTG PPK model can be accurately described on the basis of comedications influencing UGT1A4 and UGT2B7, rather than polymorphisms of *UGT1A4* and *UGT2B7*. In agreement with this idea, we screened published *UGT1A4* and *UGT2B7* polymorphisms and found that neither the *UGT1A4* nor the *UGT2B7* polymorphism was found to be a significant covariate for the CL/F of LTG in two recent studies (Smith et al., 2018; Suzuki et al., 2019). The *UGT1A4* mutant genotype at nucleotide position 142 (UGT1A4*3) is associated with a higher LTG concentration only in the presence of co-medication with VPA (Smith et al., 2018), and the effect of the UGT1A4*3 polymorphism on LTG concentration was more remarkable when the VPA concentration was higher (Liu et al., 2015). In our study, the effect of UGT polymorphisms on LTG disposition appeared to be increased in the presence of VPA, to an extent similar to that reported in previous studies (Siddiqui et al., 2003; Zhang et al., 2017; van Dijkman et al., 2018). A study-pooled analysis (van Dijkman et al., 2018) by van Dijkman has provided a recommended dose table on the basis of simulations. For both pediatric and adult patients, the LTG dose should be decreased by 50% when the therapy is combined with VPA (van Dijkman et al., 2018), in agreement with our simulated results in **Figure 4**. However, when co-administered with enzyme inducers such as carbamazepine (CBZ), oxcarbazepine, and phenobarbital, an average 0.94-fold increase in the typical CL has been observed in Chinese children with epilepsy (Zhang et al., 2017). Therefore, 60% and 138% dose increases should be considered for adult and pediatric patients, respectively, who are comedicated with CBZ (van Dijkman et al., 2018). Similarly, a 125% increase in dose was suggested for patients with concomitant RFP administration in our simulations in **Figure 4**. When the two treatments are administered concomitantly, VPA inhibition has been reported to be likely to outweigh the potential of CBZ in inducing LTG metabolism (Brzakovic et al., 2014). However, the results of our study were not in agreement with that finding: according to the final model, when VPA and RFP were administered together with LTG, the effect on the CL/F of LTG was offset. The reason for the discrepancy in the results of these studies may be that the magnitudes of metabolism induction from CBZ and RFP differed. Although no direct study has compared the induction capacity between CBZ and RFP, a quantitative measurement of urinary 6β-hydroxycortisol has shown a four-fold increase in RFP enzyme activity, whereas the extent of induction by CBZ is two- to five-fold and depends on CBZ dosage (Roots et al., 1979).

Strong but incomplete linkage disequilibrium has been detected among *MDR1* SNPs (Kim et al., 2001; Tang et al., 2002; Zheng et al., 2002; Kroetz et al., 2003). Stratification based on haplotype pair carriers could yield a stronger genotype/expression relationship. However, most PPK models of LTG do not take the effect of stratification into account and thus could yield ambiguous or conflicting results. In our data, *MDR1*-2677TT carriers were likely to have C3435T TT genotypes (91.67%); therefore, the two SNPs were treated as a combined covariate variable (*MDR1*-2677+3435). After screening the gene polymorphisms of the metabolic enzymes and transporters, the transporter polymorphisms *MDR1*-2677TT+3435TT, *ABCG2*-34AA, and *SLC22A1*-1222AA were the only genetic predictors in our final model.

The *MDR1* gene encodes a 170-kDa cellular efflux pump for xenobiotics, metabolic byproducts, and drug substrates in cancer cells and normal tissues with excretory or protective function (Kroetz et al., 2003). Although the cSNP exon 26 3435C > T does not alter the encoded Ile amino acid, the T allele probably produces an Ile codon that is infrequently utilized in the human genome and decreases translation efficiency (Tang et al., 2002). Moreover, the frequent linkage of 3435TT with 2677TT results in an A893S amino acid change in Caucasians. An *in vitro* study has demonstrated that cells carrying 2677TT show less uptake of digoxin than cells containing the major exon 21 2677G allele (Kim et al., 2001). Thus, *MDR1*-2677TT+3435TT may considerably affect the bioavailability, tissue concentration, and pharmacologic effects of many drugs. Our results demonstrated that patients with epilepsy and *MDR1*-2677TT+3435TT had a significantly higher V/F and a possibly wider distribution than non-carriers, as a result of higher steady-state trough concentrations of LTG. A similar trend has also been found in a previously published study in Chinese patients with epilepsy (Zhou et al., 2015).

The ABCG2 protein is also a multidrug pump widely expressed in the small intestine, blood–brain barrier, blood–placenta barrier, liver canalicular membranes, proximal tubule cells of the kidney, and mammary glands (Heyes et al., 2018). *ABCG2* 34G > A and *ABCG2* 421C > A both have highly variable frequencies depending on ancestry (Heyes et al., 2018). Decreased transport of the substrate drug has been observed in an *ABCG2*-34AA transfected cell line (Rudin et al., 2008). Lower *ABCG2* mRNA expression has also been detected in an *in vivo* study on liver tissues (Tamura et al., 2007), thus suggesting that the presence of the 34A allele is linked to decreased translation of the *ABCG2* gene and therefore decreased transmembrane transport of substrates. Current reports regarding the effects of *ABCG2* 34G > A in different disease areas are conflicting, and whether *ABCG2* 34G > A influences the disposition of LTG remains unclear. In our study, patients with epilepsy and *ABCG2*-34AA had significantly lower V/F, and LTG elimination was relatively easier, thus resulting in lower steady-state trough concentrations of the drug. Nevertheless, given the altered distribution in many tissues, the effect of the 34A allele on therapeutic efficacy remains ambiguous. Regarding *ABCG2* 421C > A, a decrease in overall ABCG2 protein expression has been identified in many *in vitro* and *in vivo* studies (Heyes et al., 2018). However, the current opinion on whether *ABCG2* 421C > A is a predictor of LTG concentration remains controversial. Zhou et al. (2015) and Shen et al. (2016) have observed similar associations between *ABCG2*-421AA and higher dose-normalized LTG concentrations in patients by using multiple linear regression models; in contrast, Domjanovic et al. (2018) have demonstrated that the *ABCG2* 421A allele has no effect on LTG concentrations, but the variant allele effect would increase with incremental VPA troughs. These studies have combined several simplistic situations including polymorphisms or polymorphisms plus VPA, which may not have been sufficient to distinguish and weigh multiple factors in practical use. In our final model, *ABCG2* 421C > A had no effect on LTG concentrations, similarly to the results of the Domjanovic et al. study (Domjanovic et al., 2018), whereas *ABCG2* 34G > A was found to be a significant covariate.

The uptake of LTG into hepatocytes and human brain endothelial cells is an active process mediated by SLC22A1 (Dickens et al., 2012). The 408 Met > Val in exon 7 in *SLC22A1* 1222A > G mutations probably leads to lower *OCT1* mRNA levels in the human liver (Shikata et al., 2007) and decreased transport of LTG to hepatocytes and consequently slower clearance. In the current study, carriers of the *SLC22A1*-1222AA polymorphism had a 52.5% decrease in CL and higher LTG concentrations, in agreement with findings from a recently published report (Shen et al., 2016). The higher maintenance doses of LTG in patients with the *SLC22A1*-1222AA genotype have also been identified in a systematic study (Grant, 2010).

The 2017 AGNP consensus guidelines (Hiemke et al., 2018) report therapeutic reference ranges and improved dose-related reference ranges for many neuropsychiatric drugs, providing a very useful reference for clinical practice. However, dosage adjustment procedures are not discussed, and dose interpretation is usually empirically conducted by clinicians. At least one known concentration is needed for dosage correction, and several rounds may be required to obtain satisfactory therapeutic effects for some patients. This PPK model provides a practical way to calculate the primary dosage and to determine the final dosage. Importantly, our model enables forecasting as well as prior prescription adjustment when situations change, thus potentially avoiding extreme concentration fluctuations. This genetics-based PPK model also considers comedications as well as polymorphisms in enzymes and transporters, thus providing more information about factors that influence LTG concentration and possibly enabling more accurate personalized medicine. All data in the present study were collected prospectively by pharmaceutical assistants; therefore, this method may improve the accuracy of observations without major therapy intervention. In addition, a naturalistic design, multiple observations collected over time, and a broad age range in this study increased the clinical validity of the pharmacokinetic results.

This study has some limitations. Most samples were taken at trough concentrations, whose reference ranges are provided in the AGNP guidelines; therefore, some relevant pharmacokinetic parameters, including K_a_ and the IIV of K_a_, could not be estimated. To explore the effects of fixed K_a_ values, models with two-fold or half the current fixed value of 1.97 h^−1^ (Milosheska et al., 2016) were built, and no significant influence on the estimation of CL/F and V/F was found. In addition, the reference range of LTG given in guidelines is far from the extreme high and low concentrations, thus resulting in very sparse clinical samples at these concentrations. As shown in **Figures 2** and **3**, bias in the estimation of extreme high concentrations existed, but most conditional weighted residuals (CWRES) values were still between −2 and 2. The NPDE was normally distributed and did not correlate with the time after last dose. Therefore, the current model should be suitable for most samples in clinical practice. Patients with extreme high and low concentrations of LTG under regular dosage regimens are suggested to be a special group for which, to the best our knowledge, no relevant factors contributing to extremely exceptional situations have been identified. Because such patients may have higher occurrence rates of therapeutic failure and adverse effects, we suggest they should be cautious when taking LTG. The current PPK model might not be appropriate for these patients.

Another limitation of the current study is the relatively small proportion of very young children, which limited the prediction precision of the final model for Chinese pediatric patients. Although the population model for LTG CL/F and V/F adequately illustrates the observed data, we suggest that it may be more suitable for patients 13–65 years of age. Moreover, the mutation frequency of certain genotypes may have been quite small, and these genotypes were therefore not detected in the Chinese population. Power analysis was also not conducted in our study, but we suggest that larger scale research would generate a more accurate and robust model.

## Conclusion

A population pharmacokinetic model of LTG based on sparse data adequately characterized enzyme and transporter polymorphisms in children and adults prescribed different comedications. This PPK model may prove valuable for determining personalized dose regimes in patients and may provide insight into factors affecting drug PK variability among patients.

## Data Availability

The raw data supporting the conclusions of this manuscript will be made available by the authors, without undue reservation, to any qualified researcher.

## Ethics Statement

This study was carried out in accordance with the recommendations of Declaration of Helsinki, the Medical Ethics Committee of Guangzhou Huiai Hospital with written informed consent from all subjects. All subjects gave written informed consent in accordance with the Declaration of Helsinki. The protocol was approved by the Medical Ethics Committee of Guangzhou Huiai Hospital.

## Author Contributions

DS and YW designed the research; WH, YZ, SD, XZ, CQ and JH performed the research; HL, XN, ZW, MZ, HX and HC determined the serum concentrations of lamotrigine; XW and DS built the model; and ZW, YZ and DS wrote the paper.

## Funding

The first and last funding information are inaccurate. Please change this section as follows: This work was supported by grants from the following: National Natural Science Foundation of China (Grant No 81403016), Guangzhou Municipal Psychiatric Disease Clinical Transformation Laboratory (Key Laboratory for Innovation platform Plan, Science and Technology Program of Guangzhou, Grant No. 201805010009), Guangzhou Municipal Key Discipline in Medicine (2017-2019), Medical Health Science and Technology Project of Guangzhou (Grant No. 20171A010276).

## Conflict of Interest Statement

The authors declare that the research was conducted in the absence of any commercial or financial relationships that could be construed as a potential conflict of interest.

## Supplementary Material

Supplementary Figure 1 Frequency distributions (columns, left Y-axis) and body weights (lines, right Y-axis) of different age groups in the study population of patients with epilepsy.
