# Supplementary figures and images for: Effects of Comedication and Genetic Factors on the Population Pharmacokinetics of Lamotrigine: A Prospective Analysis in Chinese Patients With Epilepsy
Source: Front Pharmacol. 2019 Jul 25;10:832. doi: 10.3389/fphar.2019.00832 (PMC6669232; doi:10.3389/fphar.2019.00832)

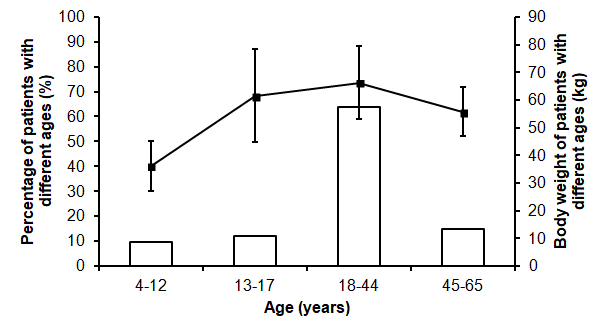

Supplement: Supplementary Figure 1 — Frequency distributions (columns, left Y-axis) and body weights (lines, right Y-axis) of different age groups in the study population of patients with epilepsy. [file Image_1.tif]
